# Formation and dissociation of synthetic hetero-double-helix complex in aqueous solutions: significant effect of water content on dynamics of structural change†

**DOI:** 10.1039/c9ra06073a

**Published:** 2019-09-18

**Authors:** Tsukasa Sawato, Ryosuke Yuzawa, Higashi Kobayashi, Nozomi Saito, Masahiko Yamaguchi

**Affiliations:** Department of Organic Chemistry, Graduate School of Pharmaceutical Sciences, Tohoku University Aoba Sendai 980-8578 Japan yama@m.tohoku.ac.jp +81-22-795-6811

## Abstract

A 1 : 1 mixture of the ethynylhelicene pseudoenantiomers (*M*)-tetramer and (*P*)-pentamer, which possess hydrophilic terminal tri(ethyleneglycol) (TEG) groups, changes their structures in the water–THF (10 μM) solvent system between dissociated random-coils and an associated hetero-double-helix upon heating and cooling. A small change in water content between 30 and 33% significantly affects the dynamics of structural changes. At 30% water content, heating to 60 °C causes rapid formation of random-coil and cooling to 10 °C causes the rapid formation of hetero-double-helix, accompanied by repeated changes in Δ*ε* at 369 nm between 0 and −2000 cm^−1^ M^−1^. Heating and cooling experiments at constant rates between 60 and 10 °C resulted in sigmoidal curves in Δ*ε*/temperature profiles, which indicate rapid structural changes. Different phenomena occurred at 33% water content. Heating to 60 °C and cooling to 0 °C initially induced changes in Δ*ε* between 0 and −2000 cm^−1^ M^−1^, and repeated cycles gradually reduced the range between 0 and −500 cm^−1^ M^−1^. Heating and cooling experiments at constant rates between 60 and 10 °C caused small changes in Δ*ε*, and repeated cycles at 10 °C gradually increased Δ*ε* to −500 cm^−1^ M^−1^. These phenomena involved rapid changes in molecular structure and slow structural changes in the water–THF solvent system. The sharp switching of the dynamics of structural changes at water content between 30 and 33% indicated discontinuous structural changes in the hydration of TEG and/or in water clusters in the vicinity of oligomer molecules.

## Abstract 2

Significant structural changes by small change in water content from 30 to 33%.

## Graphical abstract

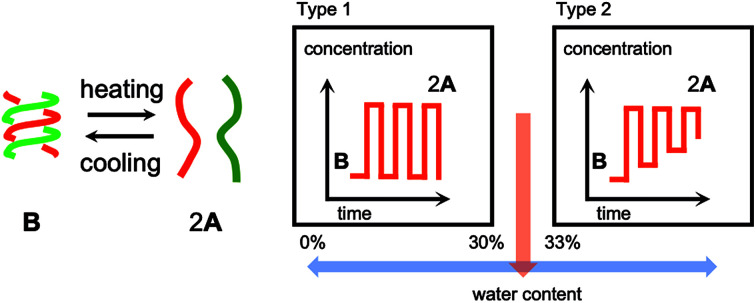

## Introduction

The hetero-double-helix, derived from linear molecules with two different structures, plays pivotal roles in biological double-stranded DNA, which dissociates and associates in water for gene regulation and expression to occur.^1,2^ Therefore, the development of hetero-double-helix from synthetic molecules in aqueous solution is attractive, because it promotes the understanding and control of such biological events as well as provides novel functional materials.^3^ Although water-soluble synthetic homo-double-helices have recently been developed^4–8^ as dimeric aggregates derived from linear molecules with the same structure, the synthesis and dynamic properties of the water-soluble hetero-double-helix have not yet been studied.

To develop a water-soluble synthetic hetero-double-helix, the presence of hydrophilic groups is essential. We previously reported that the ethynylhelicene (*M*)-tetramer (*M*)-1 with tri(ethyleneglycol) (TEG) groups formed homo-double-helix in mixed solutions of water and organic solvents.^9,10^ Association and dissociation showed an inverse thermoresponse, in which heating induced association and cooling induced dissociation. Inverse thermoresponses I and II were observed depending on the organic cosolvents used. These unusual thermoresponses were ascribed to the hydration/dehydration of the TEG groups and/or structural changes of water clusters in the vicinity of the oligomer molecules.

**Fig. 1:**
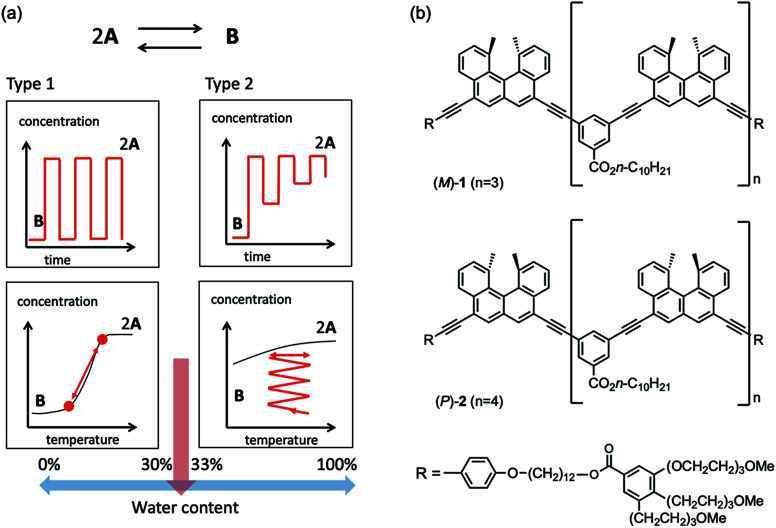
(a) Schematic presentation of dynamics of structural changes between hetero-double-helix B and random-coils 2A in water–THF solvent system, in which the changes in the water content from 30% to 33% significantly affected the dynamics, termed type I and II, respectively, as mentioned later in this paper. Red lines indicate the experimental structural change dynamics, and black lines indicate the equilibrium curves. (b) Chemical structures of (*M*)-1 and (*P*)-2.

It was also found that mixtures of the ethynylhelicene pseudoenantiomers (*M*)-oligomer and (*P*)-oligomer with different helicene numbers formed hetero-double-helix in organic solvents.^10–12^ In this study, a water-soluble hetero-double-helix was developed employing a mixture of pseudoenantiomers ethynylhelicene (*M*)-tetramer (*M*)-1 and (*P*)-pentamer (*P*)-2, which possess terminal hydrophilic TEG groups (Fig. 1b).

The (*M*)-1/(*P*)-2 mixture formed a hetero-double-helix in water-THF solvent system, which switched the dynamics of structural changes with the change in water content from 30 to 33% (Fig. 1a). At 30% water content, repeated heating to 60 °C and cooling to 10 °C changed Δ*ε* at 369 nm between 0 and −2000 cm^−1^ M^−1^, which rapidly occurred in response to temperature changes. Constant-rate heating and cooling resulted in sigmoidal curves. At 33% water content, heating to 60 °C and cooling to 10 °C initially changed Δ*ε* between −2000 and 0 cm^−1^ M^−1^, and repeated heating/cooling cycles gradually reduced the range between −500 and 0 cm^−1^ M^−1^. Heating and cooling at a constant rate resulted in smaller changes in Δ*ε*. These phenomena suggest the involvement of rapid changes in molecular structure and slow structural changes in the water–THF solvent system. The sharp switching of dynamics of structural changes caused by the small change in water content indicates a discontinuous change in the hydration/dehydration of TEG groups and/or water clusters in the vicinity of the oligomers.

## Results and discussion

### Experiments in water–THF solvent system with 30% water

**Fig. 2:**
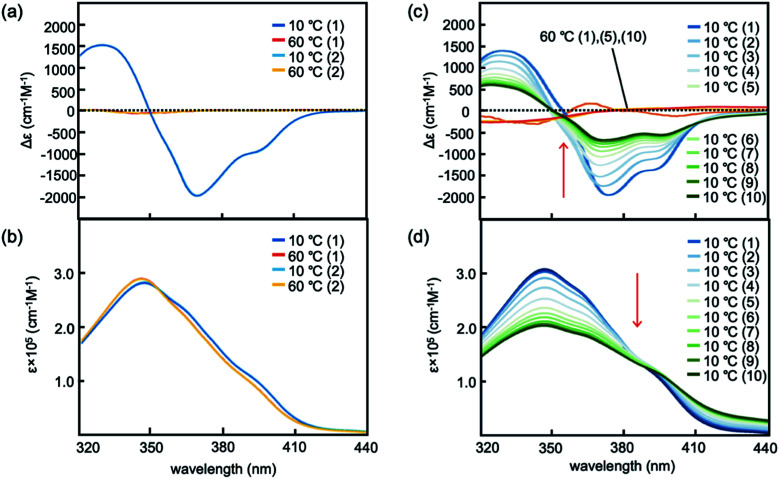
(a) CD and (b) UV-vis spectra of (*M*)-1/(*P*)-2 (1 : 1) in 30% water–THF (10 μM) at 60 and 10 °C. (c) CD and (d) UV-vis spectra of (*M*)-1/(*P*)-2 (1 : 1) in 33% water–THF (10 μM) at 60 and 10 °C. The numbers of heating/cooling cycles is shown in parentheses. See Fig. S8a† for details of the spectra in (c) and (d).

Hetero-double-helix formation by the (*M*)-1/(*P*)-2 mixture was examined using 10 μM in water-THF solvent system with 30% water, which is hereafter referred as 30% water–THF in this paper. The solution was prepared by mixing two solutions of (*M*)-1 and (*P*)-2 in 30% water–THF at 1 : 1 ratio. Heating the resulting solution to 60 °C produced dissociated random-coil 2A exhibiting a weak Cotton effect, as shown by circular dichroism (CD) spectroscopy (Fig. 2a and S1†). Cooling the solution to 10 °C showed a strong negative Cotton effect at 369 nm with Δ*ε* reaching −2000 cm^−1^ M^−1^, and the Cotton effect disappeared upon heating the solution to 60 °C. UV-vis spectra showed slightly reduced intensity upon cooling to 10 °C (Fig. 2b and S1†). These results were consistent with the structural changes between 2A and hetero-double-helix B.^9^ The experiment was also conducted using a 100 μM solution, and CD spectra obtained at 10 °C were very similar to those of the 10 μM solution (Fig. S7†), which indicated that the CD spectrum with Δ*ε* reaching −1500 cm^−1^ M^−1^ showed the equilibrium shifted to B and essentially contained no 2A. Hetero-double-helix formation by the (*M*)-1/(*P*)-2 mixture in water–THF solvent system occurred at μM-order concentrations that was much more extensive than that in organic solvents at mM-order concentrations.^11–13^ Dynamic light scattering (DLS) analysis (30% water–THF, 10 μM) at 10 and 60 °C showed the formation of particles with average diameters smaller than 10 nm (Fig. S2†). Consequently, no Tyndall effect was observed. Therefore, the thermoresponse of the (*M*)-1/(*P*)-2 mixture observed by CD spectroscopy is derived from molecules dispersed in solution that do not form larger aggregates.

**Fig. 3:**
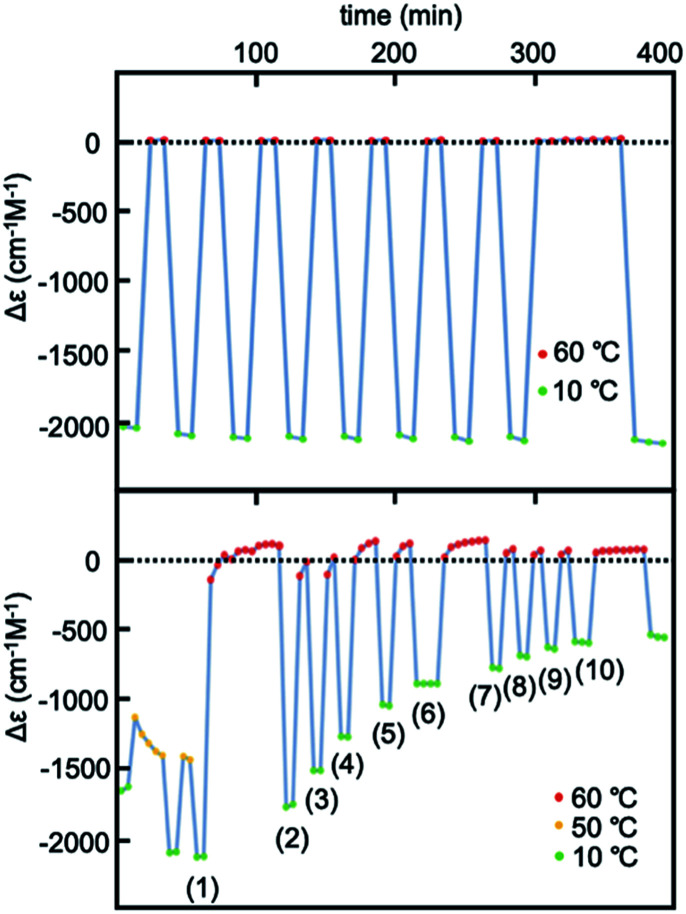
Experiments of repeated heating and cooling of (*M*)-1/(*P*)-2 (1 : 1) between 60 and 10 °C for Δ*ε* at 369 nm in (a) 30% water–THF (10 μM) and for Δ*ε* at 376 nm in (b) 33% water–THF (10 μM).

Experiments of repeated and periodic heating to 60 °C and cooling to 10 °C were conducted, and structural changes were monitored on the basis of Δ*ε*. Sharp structural changes appeared between 0 and −2000 cm^−1^ M^−1^ as shown by the rectangular shapes of Δ*ε*/time profiles (Fig. 3a and S6b†), which indicated rapid structural changes between molecules of 2A and B. Other experiments with nonperiodic temperature changes also yielded rectangular shapes of profiles (Fig. S6a†).

**Fig. 4:**
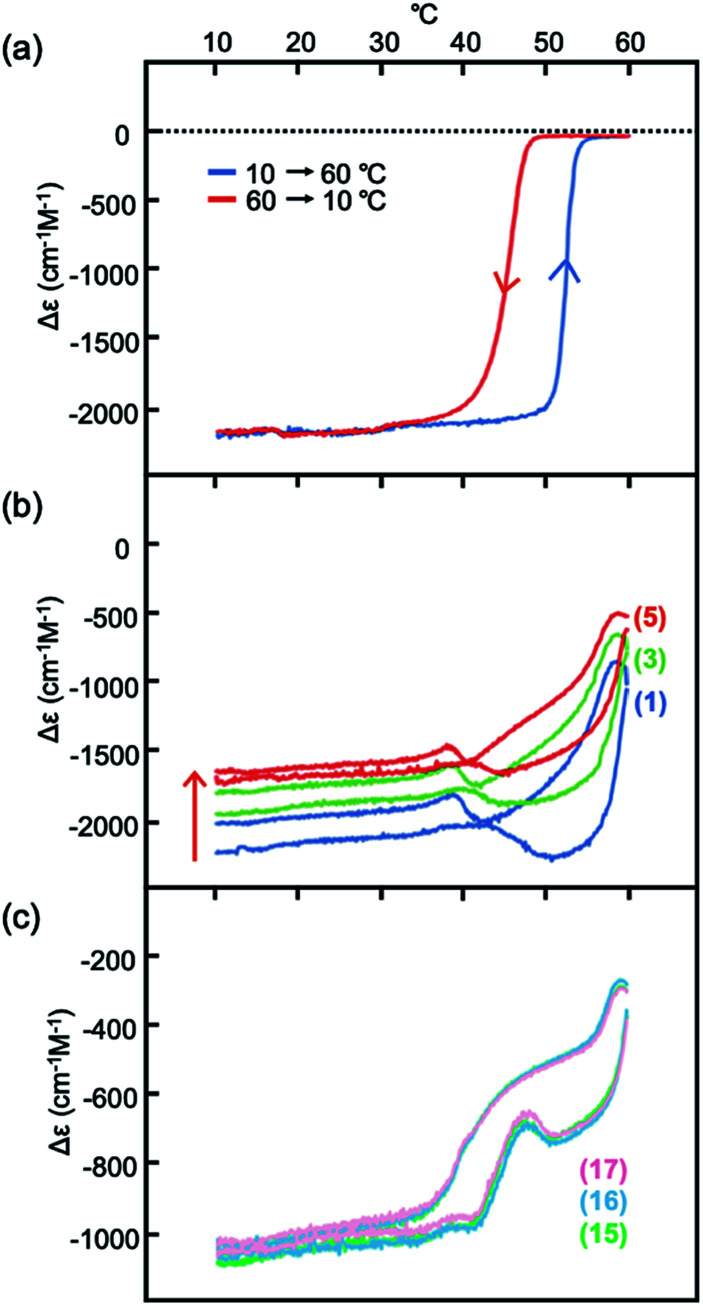
Δ*ε* (375 nm)/temperature profiles from experiments of heating and cooling of (*M*)-1/(*P*)-2 (1 : 1) at a constant rate of 1 °C min^−1^ between 10 and 60 °C, shown by (a) in 30% water–THF (10 μM); (b) in 33% water–THF (10 μM) starting from −2000 cm^−1^ M^−1^; (c) enlarged view of (b). The number of heating/cooling cycles is shown in parentheses in (b) and (c). See Fig. S17† for details.

Heating and cooling experiments at constant rate were conducted between 60 and 10 °C at a constant rate of 1 °C min^−1^ in 30% water–THF (Fig. 4a and S16†). Δ*ε* at 375 nm decreased from 0 cm^−1^ M^−1^ at 60 °C, and reached −2000 cm^−1^ M^−1^ at 10 °C. Sigmoidal curves were obtained during heating and cooling with a small thermal hysteresis, which is consistent with the observed rapid structural changes between molecules of 2A and B (Fig. 3a). These phenomena in 30% water–THF are termed type-1 behavior in this paper.

### Experiments in water–THF solvent system with 33% water

When water content was increased to 33%, different phenomena were observed. A (*M*)-1/(*P*)-2 mixture in 33% water–THF (10 μM) was prepared by mixing two solutions of (*M*)-1 and (*P*)-2 in 33% water–THF (10 μM) at 1 : 1 ratio at room temperature. The resulting mixture was heated to 50 °C and cooled to 10 °C, during which a strong negative Cotton effect was observed at 376 nm with Δ*ε* reaching −2100 cm^−1^ M^−1^ (Fig. S8a†). When heated to 50 °C, Δ*ε* increased to −1500 cm^−1^ M^−1^. Then, the solution was heated to 60 °C and cooled to 10 °C, during which Δ*ε* changed between 0 and −1700 cm^−1^ M^−1^. Repeated heating/cooling cycles gradually decreased the Δ*ε* range, and Δ*ε* at 10 °C reached −500 cm^−1^ M^−1^ after 10 cycles (Fig. 2c). UV-vis spectra also showed changes in intensity as a result of repeated heating to 60 °C and cooling to 10 °C (Fig. 2d). The structure of the (*M*)-1/(*P*)-2 mixture in 33% water–THF (10 μM) was examined by dynamic light scattering (DLS) analysis at 10, 50, and 60 °C, which showed the formation of particles smaller than 10 nm (Fig. S3†). No Tyndall effect was observed during the process, indicating that the structural changes were molecular events in solution.

Experiments of repeated heating and cooling between 50 and 10 °C showed Δ*ε* (376 nm)/time profiles indicating Δ*ε* changes between −1500 and −2200 cm^−1^ M^−1^ (Fig. 3b and S8a†). The experiments between 60 and 10 °C showed Δ*ε* changes between 0 and −1700 cm^−1^ M^−1^, which on repeated heating/cooling cycles between 60 and 10 °C gradually decreased the Δ*ε* range between 0 and −500 cm^−1^ M^−1^ after 10 cycles, and reached a steady state. Rectangular shapes of Δ*ε*/time profiles with slight deviations showed that rapid structural changes occurred between molecules of 2A and B. The gradual decrease in the Δ*ε* range with repeated heating/cooling cycles suggests a structural change of the water–THF solvent system.

Constant-rate heating and cooling experiments in 33% water–THF also exhibited dynamics of structural changes different from those in 30% water–THF. A (*M*)-1/(*P*)-2 mixture in 33% water–THF (10 μM) with Δ*ε* reaching −2000 cm^−1^ M^−1^ was obtained at 10 °C; the solution was then heated to 60 °C and cooled to 10 °C at a rate of 1 °C min^−1^ (Fig. 4b and S17†). Upon heating, Δ*ε* not change up to 45 °C, and then increased to −1000 cm^−1^ M^−1^ at 60 °C. Upon cooling, Δ*ε* decreased to −2000 cm^−1^ M^−1^ at 40 °C, and remained constant until 10 °C. The small changes in Δ*ε* in 33% water–THF were compared with large changes in 30% water–THF (Fig. 4a). Repeated heating and cooling experiments showed small changes for every cycle, and Δ*ε* at 10 °C gradually increased, reaching −1600 cm^−1^ M^−1^ after 5 cycles and −1000 cm^−1^ M^−1^ after 17 cycles (Fig. 4c). A small thermal hysteresis was observed for every cycle between 40 and 60 °C. The results of the constant-rate heating and cooling experiments in 33% water–THF indicated slow responses of Δ*ε* to temperature changes. The phenomena in 33% water–THF are referred to as type-2 behavior in the following discussions. Sharp switching in dynamics of structural changes appeared in the (*M*)-1/(*P*)-2 mixture for small changes in water content from 30% for type-1 behavior to 33% for type-2 behavior.

The effect of substrate concentration was examined in 33% water–THF. The structural changes between −1000 and 0 cm^−1^ M^−1^ appeared at 5 and 7.5 μM as well as 10 μM, which are the type-2 behavior (Fig. S9 and S10†).

Experiments were conducted in water–THF solvent system with different water contents. In 25% water–THF, heating to 60 °C and cooling to 10 °C showed structural changes between Δ*ε* 0 and –2300 cm^−1^ M^−1^, which is the type-1 behavior (Fig. S5 and S15†). In 40% water–THF, heating to 50 °C and cooling to 10 °C showed structural changes between Δ*ε* −1200 and −1700 cm^−1^ M^−1^, which changed between −200 and −1200 cm^−1^ M^−1^ upon heating to 60 °C and cooling to 10 °C; these changes are the type-2 behavior (Fig. S11†). Constant-rate temperature change experiments showed similar Δ*ε*/temperature profiles of 40% water–THF to those of 33% water–THF (Fig. S18†). An important role of water in the type-1 and type-2 behaviors was confirmed by experiments in THF without water, in which dissociated 2A persisted without formation of B upon heating and cooling (Fig. S4†). These results indicate a sharp switching in dynamics of the structural changes of the (*M*)-1/(*P*)-2 mixture at water contents between 30 and 33% owing to the type-1 and -2 behaviors.

### Experiments involving changes in water content

**Fig. 5:**
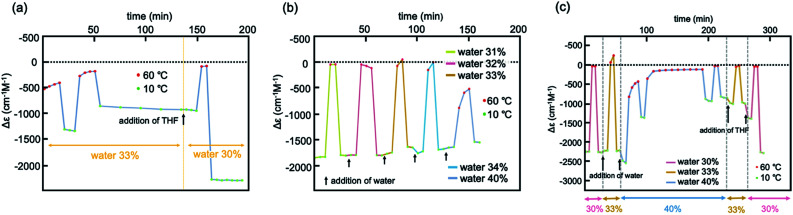
Experiments using (*M*)-1/(*P*)-2 (1 : 1) with change in water content in water–THF (10 μM) at 371 nm. (a) From 33% to 30% by adding THF; (b) from 30% to 31, 32, 33, and 34% by adding water; (c) from 30% to 33%, and 40% by adding water, and then to 33% by adding THF.

In the above experiments, the (*M*)-1/(*P*)-2 mixture in 30% water–THF (10 μM) was prepared by mixing two solutions of (*M*)-1 and (*P*)-2 in 30% water–THF. Another procedure was examined, in which the water content was changed from 33% to 30% in a single vessel by adding THF to 33% water–THF. A (*M*)-1/(*P*)-2 mixture in 33% water–THF (10 μM) was prepared by mixing two solutions of (*M*)-1 and (*P*)-2 in 33% water–THF, and the resulting solution showed structural changes between −300 and −1300 cm^−1^ M^−1^ upon heating to 60 °C and cooling to 10 °C, which is the type-2 behavior (Fig. 5a and S12†). THF was added to prepare a 30% water–THF solution, and the resulting solution showed structural changes between −2500 and 0 cm^−1^ M^−1^, which is the type-1 behavior. Rectangular shapes of structural changes in Δ*ε*/time profiles are consistent with rapid structural changes between molecules of 2A and B. These results confirmed the sharp switching of dynamics of the structural changes of the (*M*)-1/(*P*)-2 mixture caused by changing the water content from 33 to 30%.

A reverse experiment, in which 30% water–THF (10 μM) was changed to 33% water–THF in a single vessel by adding water, showed different phenomena. A (*M*)-1/(*P*)-2 mixture in 30% water–THF was prepared by mixing two solutions of (*M*)-1 and (*P*)-2 in 30% water–THF; this showed structural changes between −1800 and 0 cm^−1^ M^−1^ upon cooling to 10 °C and heating to 60 °C, as the type-1 behavior (Fig. 5b and S13†). Water was added to obtain 31% water–THF solution, which also showed structural changes between −1800 and 0 cm^−1^ M^−1^ (type-1 behavior). The type-1 behavior also appeared in 32, 33, and 34% water–THF upon addition of portions of water. Simply increasing of the water content from 30% to 33% did not result in a switch from the type-1 to type-2 behavior. Rectangular shapes associated with structural changes appearing in Δ*ε*/time profiles are consistent with rapid structural changes of molecules of (*M*)-1 and (*P*)-2.

It was observed that the type-2 behavior appeared as a result of addition of THF to 40% water–THF solution (Fig. 5c and S14†). Water was added to (*M*)-1/(*P*)-2 mixture in 30% water–THF to prepare 40% water–THF, which showed structural changes between −1000 and 0 cm^−1^ M^−1^ (type-2 behavior). THF was added to prepare 33% water–THF, and the structural changes between −1000 and 0 cm^−1^ M^−1^ was observed, which are the type-2 behavior. To switch from the type-1 to type-2 behavior, it is essential to employ complex procedures involving the addition of water to 30% water–THF to prepare 40% water–THF and the addition of THF to prepare 33% water–THF. The phenomenon is consistent with slow structural changes in the water–THF solvent system as a result of changing the water content from 30 to 33%.

### Mechanistic model

**Fig. 6:**
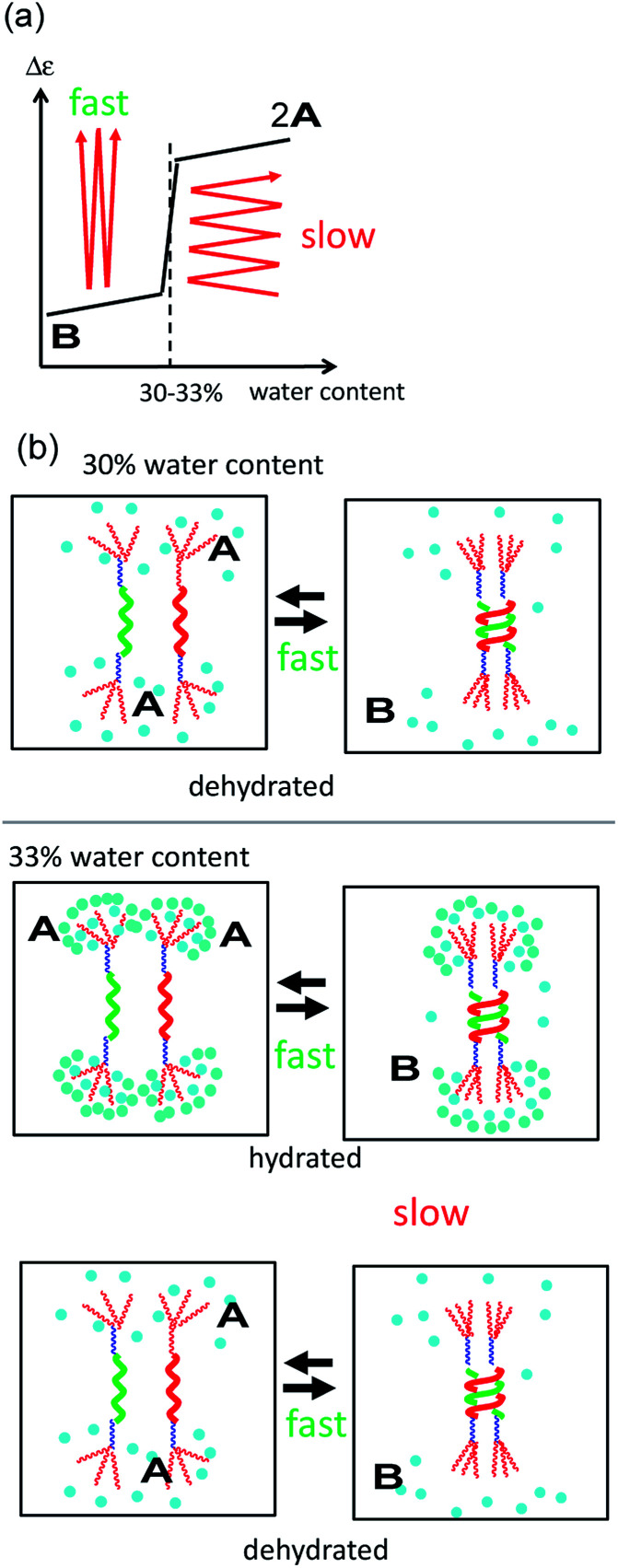
(a) Mechanistic model of dynamics in the structural changes shown by Δ*ε*/water content profiles in 30% water–THF (type-1 behavior) and 33% water–THF (type-2 behavior). Red arrows indicate structural changes between random-coil 2A and hetero-double-helix B. Black lines show the equilibrium state at 10 °C, which switches between 30 and 33% water–THF. (b) Mechanistic model of type-1 and type-2 behaviors. In the type-1 behavior, rapid molecular structural changes between molecules of 2A and B occur upon heating and cooling, which are shown by black arrows. In the type-2 behavior, rapid structural changes between molecules of 2A and B occur upon heating and cooling, and slow structural changes of the water–THF solvent system occur. Repeated heating and cooling slowly changes the structure of the solvent system, which is shown by bold dark red arrows. Blue circles indicate water molecules, which undergo slow changes in their hydrated/dehydrated state and/or water cluster structure.

A mechanism underlying the different dynamics of structural changes between 2A and B in 30 and 33% water–THF defined as the type-1 and type-2 behaviors, respectively, is proposed, in which the dynamics of structural changes between molecules of 2A and B and the water–THF solvent system were separately considered (Fig. 6). Structural changes of molecules of 2A and B are rapid both in 30% and 33% water–THF solutions (Fig. 6b, black arrows), which are shown by rectangular shapes and small thermal hysteresis (Fig. 3 and 4). The dynamics of slow structural changes of 33% water–THF was ascribed to the slow structural changes of the solvent system. In 30% water–THF, the equilibrium state at 10 °C is close to Δ*ε* −2000 cm^−1^ M^−1^ and at 0 cm^−1^ M^−1^ at 60 °C, and the structural changes are fast (Fig. 6a). In 33% water–THF, the Δ*ε* range gradually narrowed after repeated heating/cooling cycles from 0 and −2000 cm^−1^ M^−1^ to 0 and −500 cm^−1^ M^−1^, which can be ascribed to the slow structural changes of the 33% water–THF (Fig. 6b). Repeated heating and cooling is required to approach the equilibrium state of in the 33% water–THF. The equilibrium state at 10 °C may be close to −500 cm^−1^ M^−1^. Thus, structural changes of molecules of 2A and B are rapid in 30 and 33% water–THF, and structural changes of the 33% water–THF solvent system are slow. Such sharp switching of structural changes of (*M*)-1/(*P*)-2 mixtures can be used to sense subtle environmental changes.

The change in the structure of the solvent system can be described by the changes in the hydrated/dehydrated state of the TEG groups and/or by the changes in the structure of water clusters. In water–THF solvent system with water content below 30%, hetero-double-helix formation is rapid in response to temperature changes, because the TEG groups are less hydrated. In water–THF solvent system with water content above 33%, interconversion between molecules of 2A and B is rapid, and the hydration–dehydration of TEG groups becomes slow. Alternatively, the structure of water clusters in the vicinity of the molecules of B changes depending on the water content, which slowly responds to temperature changes.

## Conclusions

The nature of homo- and hetero-double-helix derived from TEG-containing ethynylhelicene oligomers was compared in water–THF solvent system at 10 μM. A notable difference is the ordinary thermoresponse of hetero-double-helix as observed in this study and the inverse thermoresponses of homo-double-helix as described previously.^9,10^ It is considered that this difference may be derived from the hydration/dehydration structures of the TEG groups and/or water cluster structures in the vicinity of oligomer molecules. The effect of water content on structural changes of homo- and hetero-double-helix complexes in this study is also notable. Between 30 and 33% water contents, the interconversion between 2A and B showed significant switching in dynamics of the structural changes, as described by the type-1 and type-2 behaviors. It was previously observed that a homo-double-helix formed by (*P*)-1 showed significant switching in dynamics of structural changes at water contents below and above 30% in water–THF solvent system, which switched from ordinary thermoresponse to inverse thermoresponse II.^10^ Thus, the nature of structural changes derived from hydration/dehydration of TEG groups and/or water clusters in the vicinity of the oligomer molecules appears to change at water contents of approximately 30%.

Mixed solvent systems of water and THF have attracted interest, and the dependence of their thermodynamic properties as a function of water content has been studied. Changes in the properties of water–THF solvent systems appeared at 70% water content, as shown by viscosity, X-ray scattering, molecular aggregation, and fluorescence analyses.^14–19^ In contrast, a change at 30% water content was observed in this study and in the inverse thermoresponse II,^10^ which can partly be due to the nature of interactions between TEG groups and water clusters.

To summarize, a 1 : 1 mixture of the ethynylhelicene pseudoenantiomers, (*M*)-tetramer and (*P*)-pentamer, both of which possess hydrophilic terminal TEG groups, formed hetero-double-helix and random-coils in water–THF solvent system at 10 μM. A small change in water content from 30 to 33% significantly affected dynamics of the structural changes. In 30% water–THF, heating to 60 °C caused the dissociation of hetero-double-helix to random-coils, and cooling to 10 °C caused the formation of hetero-double-helix, which rapidly and repeatedly changed Δ*ε* between 0 and −2000 cm^−1^ M^−1^. Constant-rate heating and cooling experiment showed rapid changes in Δ*ε*. In 33% water–THF, heating to 60 °C and cooling to 10 °C showed changes in Δ*ε* between 0 and −2000 cm^−1^ M^−1^, and repeated heating and cooling experiments gradually widened the Δ*ε* range to 0 and −500 cm^−1^ M^−1^. Constant-rate heating and cooling experiments showed small changes in Δ*ε*, and Δ*ε* at 10 °C gradually increased. The dynamics in the structural changes indicated slow changes of the water–THF solvent system upon heating and cooling. Sharp switching of reaction mechanism between 30 and 33% water contents suggests discontinuous structural changes in TEG hydration/dehydration and/or water clusters in the vicinity of the oligomers in the water–THF solvent system. Water is a complex molecular system,^20^ the structure of which sensitively and significantly affects molecules depending on the structures of solutes and the water content in mixed solvent systems.

## Conflicts of interest

There are no conflicts to declare.

## Supplementary Material

RA-009-C9RA06073A-s001

## References

[cit1] D Voet D. and Voet J. G., Biochemistry, Wiley, New Jersey, 4th edn, 2011

[cit2] Lodish H. , Berk A., Kaiser C. A., Krieger M., Scott M. P., Bretscher A., Ploegh H., and Matsudaira P., Molecular Cell Biology, W. H. Freeman and Company, New York, 6th edn, 2008

[cit3] Yashima E., Ousaka N., Taura D., Shimomura K., Ikai T., Maeda K. (2016). Chem. Rev..

[cit4] de Hatten X., Asil D., Friend R. H., Nitschke J. R. (2012). J. Am. Chem. Soc..

[cit5] Goto H., Katagiri H., Furusho Y., Yashima E. (2006). J. Am. Chem. Soc..

[cit6] Shang J., Gan Q., Dawson S. J., Rosu F., Jiang H., Ferrand Y., Huc I. (2014). Org. Lett..

[cit7] Goto H., Furusho Y., Yashima E. (2007). J. Am. Chem. Soc..

[cit8] Ben T., Furusho Y., Goto H., Miwa K., Yashima E. (2009). Org. Biomol. Chem..

[cit9] Saito N., Kobayashi H., Yamaguchi M. (2016). Chem. Sci..

[cit10] Saito N., Kobayashi H., Yamaguchi M. (2017). Tetrahedron.

[cit11] Shigeno M., Kushida Y., Yamaguchi M. (2015). ChemPhysChem.

[cit12] Shigeno M., Kushida Y., Yamaguchi M. (2016). Chem. Commun..

[cit13] Saito N., Yamaguchi M. (2018). Molecules.

[cit14] Takamuku T., Nakamizo A., Tabata M., Yoshida K., Yamaguchi T., Otomo T. (2003). J. Mol. Liq..

[cit15] Nayak J. N., Aralaguppi M. I., Naidu B. V. K., Aminabhavi T. M. (2004). J. Chem. Eng. Data.

[cit16] Katayama M., Ozutsumi K. (2008). J. Solution Chem..

[cit17] Feng X., Tong B., Shen J., Shi J., Han T., Chen L., Zhi J., Lu P., Ma Y., Dong Y. (2010). J. Phys. Chem..

[cit18] Marcus Y. (2012). J. Mol. Liq..

[cit19] Indra S., Guchhait B., Biswas R. (2016). J. Chem. Phys..

[cit20] Brini E., Fennell C. J., Fernandez-Serra M., Hribar-Lee B., Luksic M., Dill K. A. (2017). Chem. Rev..

