# Supplementary material for: Formation and dissociation of synthetic hetero-double-helix complex in aqueous solutions: significant effect of water content on dynamics of structural change
Source: RSC Adv. 2019 Sep 18;9(50):29456–62. doi: 10.1039/c9ra06073a (PMC9071914; doi:10.1039/c9ra06073a)
Supplement: RA-009-C9RA06073A-s001 [file RA-009-C9RA06073A-s001.pdf]

Electronic Supplementary Material (ESI) for RSC Advances.
This journal is © The Royal Society of Chemistry 2019

Formation and Dissociation of Synthetic Hetero-double-helix Complex in Aqueous Solutions:
Significant Effect of Water Content on Dynamics of Structural Change

Tsukasa Sawato, Ryosuke Yuzawa, Higashi Kobayashi, Nozomi Saito, and Masahiko Yamaguchi*

Department of Organic Chemistry, Graduate School of Pharmaceutical Sciences,

Tohoku University, Aoba, Sendai 980-8578, Japan

*E-mail: yama@m.tohoku.ac.jp, Fax: (+81) 22-795-6811.

Supporting Information

Table of Contents

1. Synthesis
2. Hetero-double-helix formation
3. Hetero-double-helix formation under different water content conditions
4. Change of water content from 33% to 30%
5. Constant rate temperature change experiment
6. References

<General method>

Melting points were determined with a Yanaco micro melting point apparatus without correction. Elemental analyses were conducted with a Yanaco CHN CORDER MT-6. Optical rotations were measured on a JASCO DIP-340 digital polarimeter. IR spectra were measured on a JASCO FT/IR-400 spectrophotometer. CD spectra were measured on a JASCO J-720 spectropolarimeter. UV-Vis spectra were measured on a BECKMAN DU 640 or a JASCO J-720 spectropolarimeter. ^{1}H NMR (400 MHz) spectra were recorded on a Varian Vnmr J2.2C or a JEOL JNM-ECA 600 (600 MHz) with

tetramethylsilane as an internal standard. ^{13}C NMR spectra were recorded on a Varian Vnmr J2.2C (100 MHz) or a JEOL JNM-ECA 600 (150 MHz) and were referenced to the solvent of $CDCl_3$ (δ 77.0, t). Chemical shifts are expressed in parts per million (ppm, δ). The abbreviations of signal patterns are as follows: s, singlet; d, doublet; t, triplet; q, quartet; quin, quintet; m, multiplet. Low- and High- resolution mass spectra were recorded on a JEOL JMS-DX-303, a JMS-AX-500, or a JEOL JMS-700. MALDI-TOF MS spectra were recorded on a Shimadzu AXIMA Assurance using α-cyano-4-hydroxycinnamic acid as the matrix. Vapor pressure osmometry (VPO) was conducted with KNAUER K-7000 molecular weight apparatus using benzil as a standard. 918 (Japan Analytical Industry, Co. Ltd.). Dynamic light scattering determined at 173° scattering angle were observed by a Zetasizer Nano S90. Atomic force microscopic (AFM) images were obtained on a Veeco Digital Instrument Multimode Nanoscope IIIa in the tapping mode regime under ambient temperature and pressure. Micro-fabricated silicon cantilever tips (Olympus OMCL-AC160TS-C2) were used. Polarized optical microscopic observation (POM) were carried out with an Olympus BX53 optical polarizing microscope equipped with a microscope digital camera Olympus DP21, a Linkam 10083 hot stage, and a freezing stage. Temperature dependent small-angle X-ray scattering (SAXS) measurements were carried out using a Rigaku Nano-viewer system equipped with a Mettler FP82 HT hot stage. Gel permeation chromatography (GPC) was conducted with Recycling Preparative HPLC LC-908 or LC-918 (Japan Analytical Industry, Co. Ltd.). CD and UV-Vis spectra were recorded using distilled or spectrophotomeric grade commercial solvents. Solvents used for AFM and POM and DLS studies were used after filtration through 0.2 μm pore membrane filters.

1. Synthesis

Ethynylhelicene (*P*)-pentamer (*P*)-**2** with TEG terminal groups was synthesized as described before.[S1] Ethynylhelicene (*M*)-tetramer (*M*)-**1** with TEG terminal groups was synthesized from ethynylhelicene (*M*)-tetramer[S2] as follows.

(*P*)-Ethynylhelicene pentamer with TEG terminal groups (*P*)-2 Under an argon atmosphere, a mixture of 3,4,5-tris{2-[2-(2-methoxy)ethoxy]ethoxy}benzoic acid 12-(4-iodophenoxy)dodecyl ester (44.8 mg, 0.045 mmol), tris(dibenzylideneacetone)dipalladium(0) chloroform adduct (4.89mg, 4.5 μmol), cuprous iodide (10.3 mg, 0.054mmol), tris(2,4,6-trimethylphenyl)phosphine (10.5 mg, 0.027 mmol), triphenylphosphine (7.08 mg, 0.027 mmol), tetrabutylammonium iodide (133.0 mg, 0.36 mmol), triethylamine (0.3 mL), and *N,N*-dimethylformamide (3.0 mL) was freeze-evacuated three times in flask A. In flask B, a mixture of ethynylhelicene (*P*)-pentamer (38.3 mg, 0.015 mmol) in THF (3.0 mL) was freeze-evacuated three times, and the mixture was slowly added to flask A. The mixture was stirred at 25℃ for 1 h. The reaction was quenched by adding saturated aqueous

ammonium chloride, and the organic materials were extracted with ethyl acetate. The organic layer was washed with brine, and dried over sodium sulfate. The solvents were evaporated under reduced pressure, and separation by silica gel chromatography and recycling GPC gave (*M*)-**1** as orange solid (51.1 mg, 0.012 mmol, 81.0%). Mp 68-70 °C ($CHCl_3$-hexane). $[\alpha]_D^{30}$ -291 (c 1.0, chloroform). MALDI-TOF-MS m/z Calcd for $C_{280}H_{316}O_{38}$: 4289.6. Found: 4289.0. IR (neat) 2954, 1720, 1595, 1509, 1244, 1113, 766 cm^{-1}. Anal. ($C_{280}H_{316}O_{38}$). Calcd: C, 78.40; H, 7.43. Found: C, 78.39; H, 7.40. ^{1}H NMR (400 MHz, $CDCl_3$) δ 0.85 (12H, t, *J* = 6.8 Hz), 1.26-1.50 (88H, m), 1.72-1.88 (16H, m), 1.96-2.00 (30H, m), 3.37 (18H, s), 3.53 (12H, m), 3.65 (24H, m), 3.73 (12H, m), 3.80 (4H, t, *J* = 5.0 Hz), 3.87 (8H, t, *J* = 5.0 Hz), 4.00 (4H, t, *J* = 6.6 Hz), 4.21 (12H, m), 4.28 (4H, t, *J* = 6.8 Hz), 4.41 (4H, t, *J* = 6.6 Hz), 4.42 (4H, t, *J* = 6.8 Hz), 6.94 (4H, d, *J* = 8.8 Hz), 7.29 (4H, s), 7.47 (6H, t, *J* = 7.2 Hz), 7.50 (4H, d, *J* = 7.2 Hz), 7.62-7.76 (14H, m), 8.03 (2H, s), 8.12 (8H, m), 8.20 (4H, dt, J = 6.8, 1.6 Hz), 8.36 (8H, m), 8.55 (10H, m). ^{13}CNMR (100 MHz, $CDCl_3$) δ 14.1, 22.6, 23.2, 25.98, 26.02, 28.71, 29.20, 29.30, 29.41, 29.54, 29.57, 31.9, 58.99, 59.01, 65.2, 65.8, 68.1, 68.8, 69.6, 70.49, 70.53, 70.65, 70.77, 71.88, 72.35, 86.2, 89.24, 89.28, 89.40, 92.8, 93.0, 95.1, 108.9, 114.6, 115.1, 119.6, 119.79, 119.81, 120.9, 123.5, 123.6, 123.7, 124.24, 124.26, 124.31, 125.3, 126.2, 126.7, 126.8, 127.0, 128.8, 129.1, 129.2, 129.3, 129.8, 129.9, 130.95, 131.00, 131.02, 131.2, 131.41, 131.43, 131.9, 132.2, 132.3, 133.1, 136.75, 136.80, 136.92, 138.25, 138.29, 142.4, 152.2, 159.4, 165.4, 166.2.

2. Hetero-double-helix formation

<CD and UV-vis analyses>

Solutions of (*M*)-**1** and (*P*)-**2** in 30% water-THF (10 μM) were prepared, and were mixed in 1:1 ratio. The resulted solution was heated at 60 °C for 10 min to form a dissociated random-coil 2**A** state, and CD and UV-vis analyses were conducted. The solution was cooled to 10 °C, and CD and UV-vis analyses were conducted for hetero-double-helix **B** solution (Figure S1).

(a) (b)

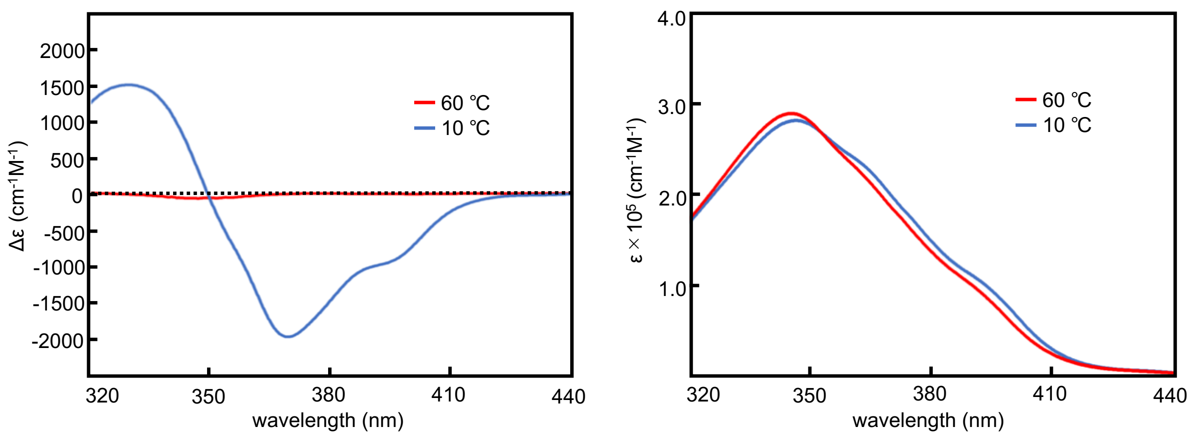

Figure S1. (a) CD and (b) UV-vis spectra of hetero-double-helix (*M*)-**1**/(*P*)-**2** (1:1) in 30% water-THF (10 μM) at 10 and 60 °C. These are identical with Figures 2a and 2b.

<DLS analysis in 30% water-THF>

A solution of (*M*)-**1**/(*P*)-**2** (1:1) in 30% water-THF (10 μM) was heated at 60 °C for 10 min to form a 2**A** solution, and was cooled to 10 °C. DLS analysis was conducted with the scattering angle of 173° (Figure S2).

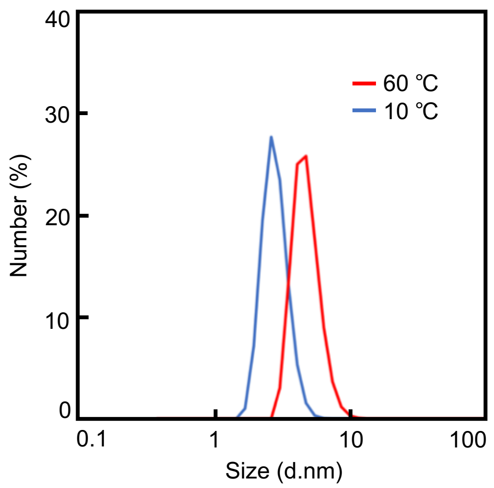

Figure S2. Number average size distributions determined by DLS of (*M*)-**1**/(*P*)-**2** (1:1) in 30% water-THF (10 μM) at 60 and 10 °C.

<DLS analysis in 33% water-THF>

Solutions of (*M*)-**1** and (*P*)-**2** in 33% water-THF (10 μM) were prepared, and were mixed in 1:1 ratio. The resulted solution was heated at 60 °C for 10 min to form a 2**A** state. The solution was cooled to 60, 50, and 10 °C in this order (Figures S3a, S3b, and S3c), and DLS analysis was conducted with the scattering angle of 173° (Figure S3). Procedures of hearing at 60 °C and cooling to 10 °C were repeated, and DLS analysis was conducted at 10 °C (Figure S3c).

(a) (b)

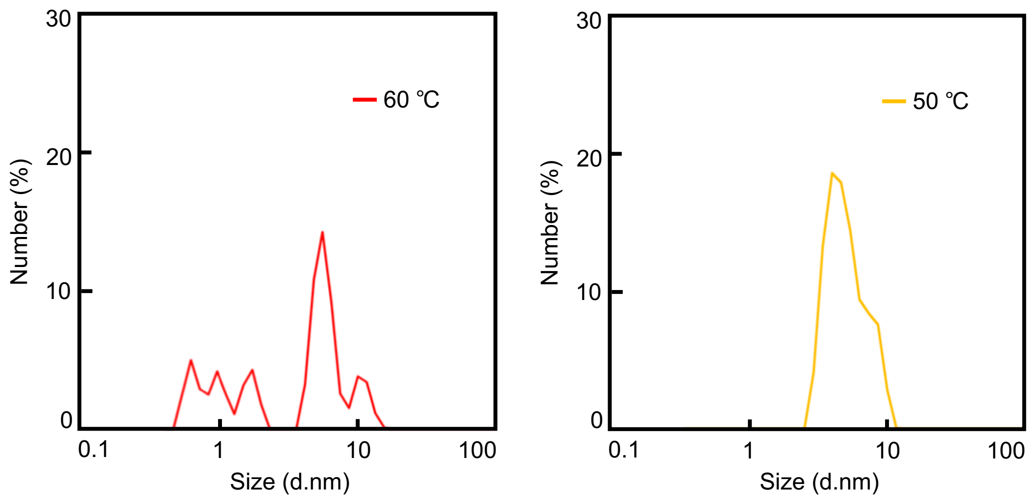

(c)

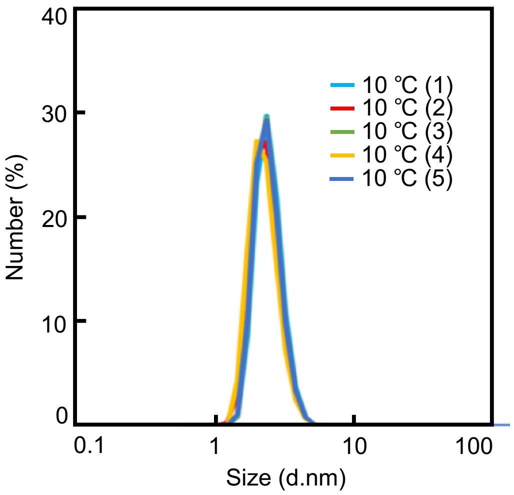

Figure S3. Number average size distributions determined by DLS of (*M*)-**1**/(*P*)-**2** (1:1) in 33% water-THF (10 μM) at 60 °C (a), 50 °C (b), and 10 °C (c). The analysis at 10 °C by five heating and cooling procedures is also shown in (c).

3. Structure changes under different water content conditions

Solutions of (*M*)-**1** and (*P*)-**2** in water-THF (10 μM) with different water contents were prepared, and were mixed in 1:1 ratio. The resulted solutions were heated at 60 °C for 10 min to form 2**A** states. Then, the solutions were cooled and heated as shown in the following Figures, and were analyzed by CD and UV-vis.

The Δε/time profiles were obtained by changing the temperature and settling the solution at the temperature shown in Figures.

<THF solution>

(a) (b)

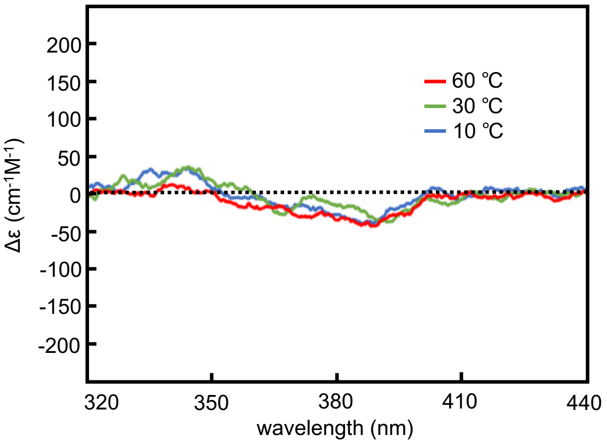

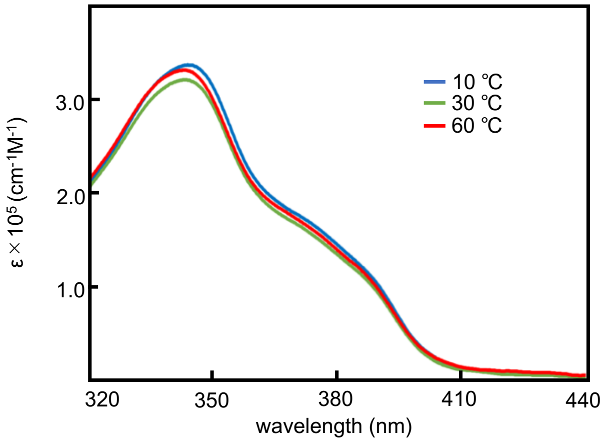

Figure S4. (a) CD and (b) UV-vis spectra of (*M*)-**1**/(*P*)-**2** (1:1) in THF (10 μM) at 60, 30 and 10 °C.

<25% water-THF solution>

(a) (b)

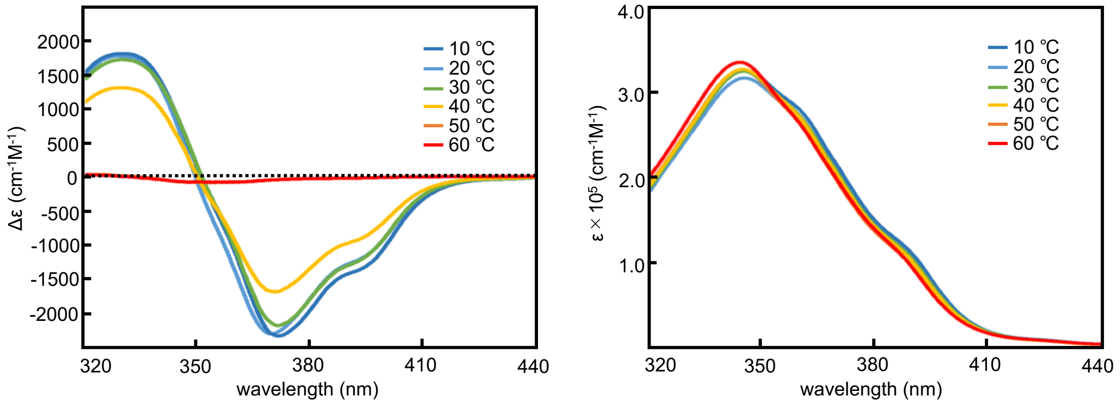

Figure S5. (a) CD and (b) UV-vis spectra of (*M*)-**1**/(*P*)-**2** (1:1) in 25 %water-THF (10 μM) at different temperatures.

<30% water-THF solution>

(a) (b)

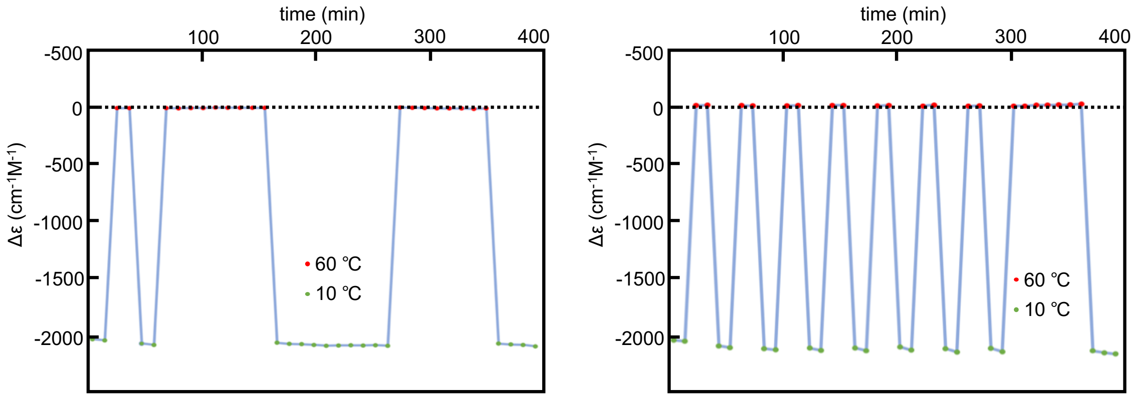

Figure S6. (a) The Δε (369 nm)/time profiles of (*M*)-**1**/(*P*)-**2** (1:1) in 30% water-THF (10 μM) by changing the temperature between 10 and 60 °C at different intervals. (b) is identical with Figure 3a.

(a) (b)

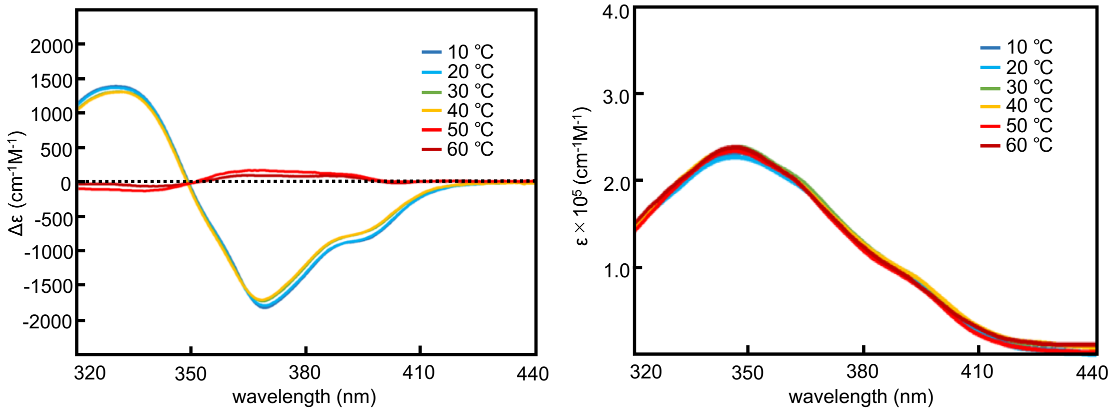

Figure S7. (a) CD and (b) UV-vis spectra of (*M*)-**1**/(*P*)-**2** (1:1) in 30% water-THF (100 μM) at

different temperatures.

<33% water-THF solution>

(a)

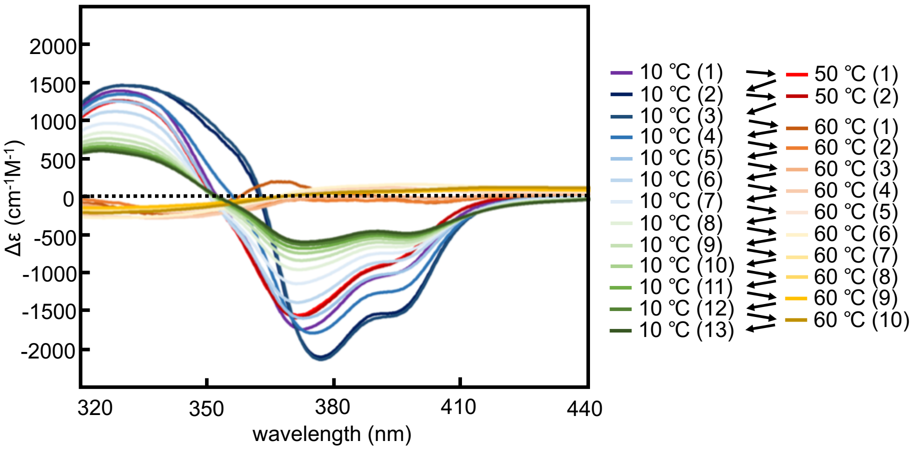

(b)

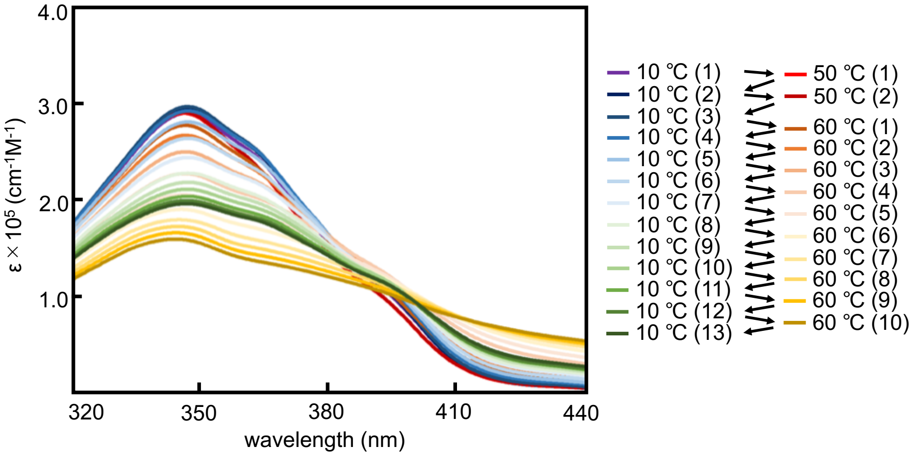

(c)

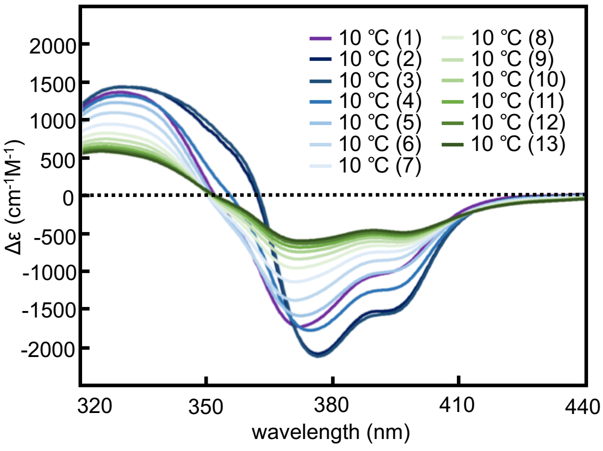

(d)

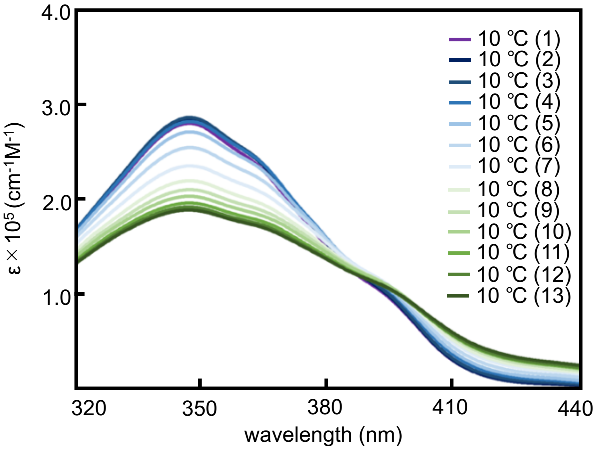

(e)

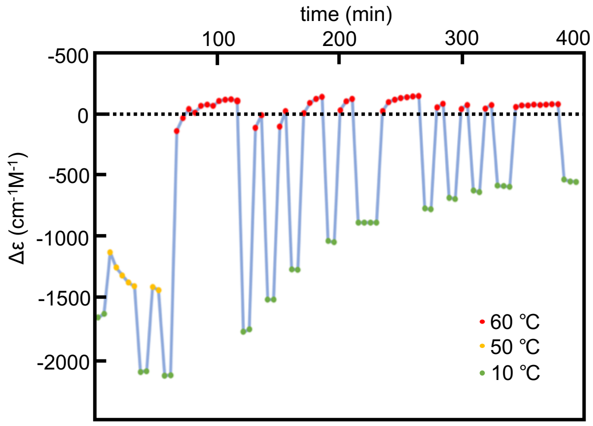

Figure S8. (a) CD and (b) UV-vis spectra of (*M*)-**1**/(*P*)-**2** (1:1) in 33% water-THF (10 μM) under repeated experiment heating to 50/60 °C and cooling to 10 °C. The numbers of heating and cooling cycles are shown in parentheses. (c) Part of Figure S8a. (d) Part of Figure S8b. (e) The Δε (376 nm)/time profiles is identical with Figure 3b.

(a) (b)

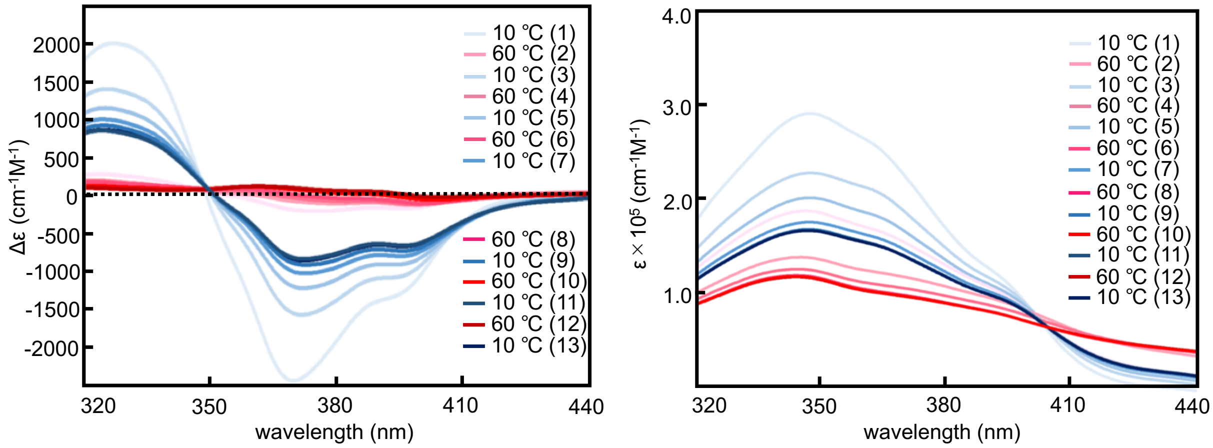

Figure S9. (a) CD and (b) UV-vis spectra of (*M*)-**1**/(*P*)-**2** (1:1) in 33% water-THF (7.5 μM) at different temperatures.

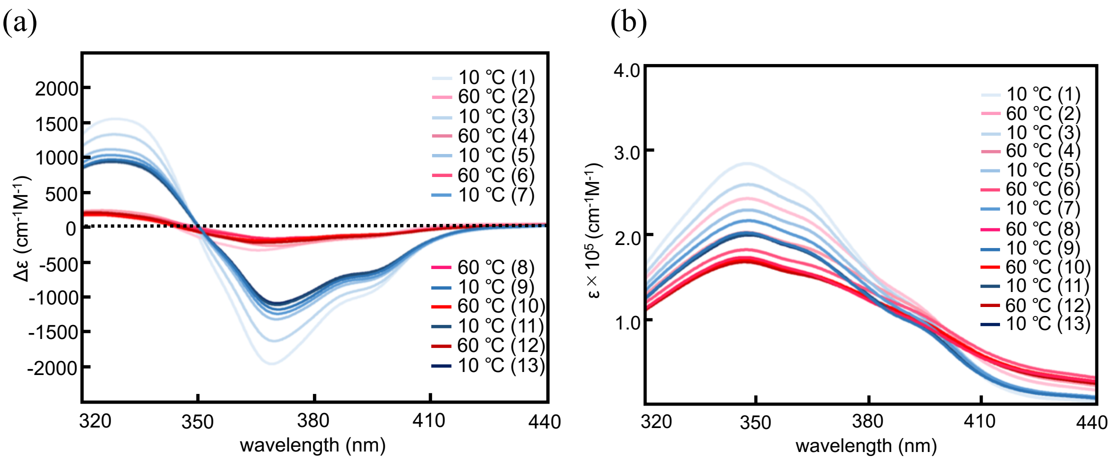

Figure S10. (a) CD and (b) UV-vis spectra of (*M*)-**1**/(*P*)-**2** (1:1) in 33% water-THF (5 μM) at different temperatures.

<40% water-THF solution>

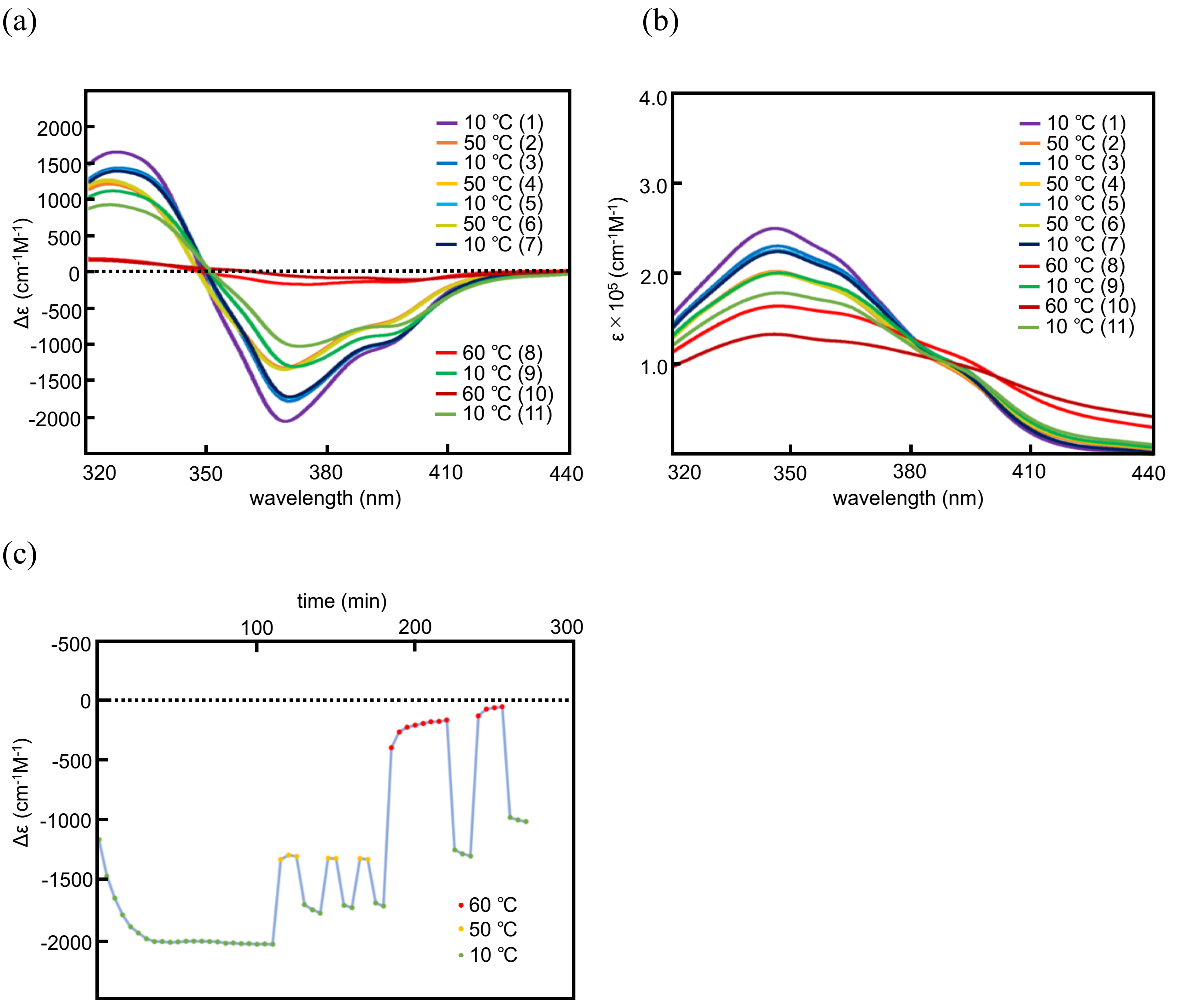

Figure S11. (a) CD and (b) UV-vis spectra of (*M*)-**1**/(*P*)-**2** (1:1) in 40% water-THF (10 μM) at different temperatures. The numbers of heating and cooling cycles are shown in parentheses. (c) The Δε (370 nm)/time profiles are shown by changing the temperature between 10 and 50 °C, which was followed by changing between 10 and 60 °C.

4. Change of water content between 33% and 30%

<From 33% to 30% experiment>

Solutions of (*M*)-**1** and (*P*)-**2** in 33% water-THF (10 μM) were prepared, and were mixed in 1:1 ratio. The resulted solution was heated at 60 °C for 10 min to form a 2**A** state, cooled to 10 °C, and heated as shown in Figure S12. Then, THF was added to adjust the water content to 30%, and the solution was heated and cooled, which was analyzed by CD and UV-vis (Figures S12(a) and S12(b)). The Δε (371 nm)/time profiles were obtained by changing the temperature and settling the solution at the temperature.

(a)

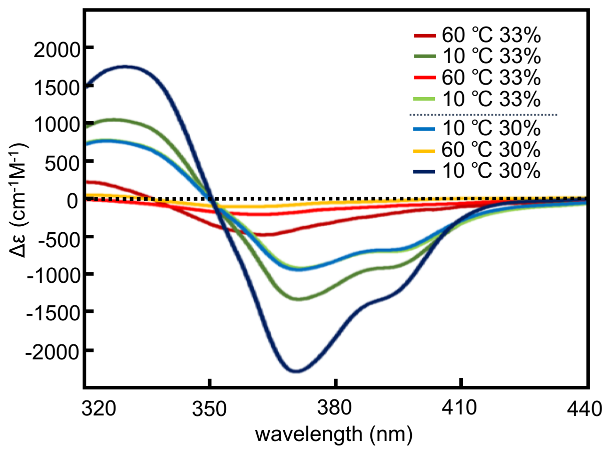

(b)

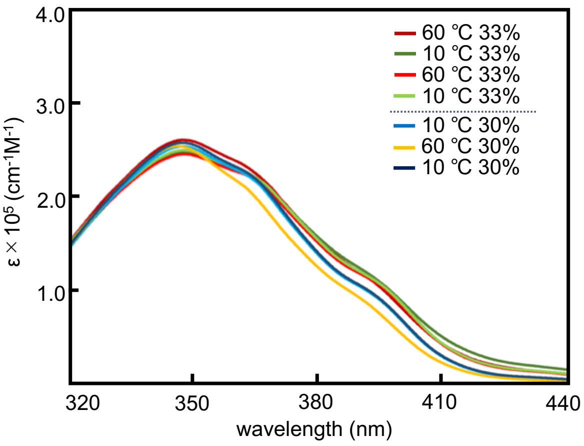

(c)

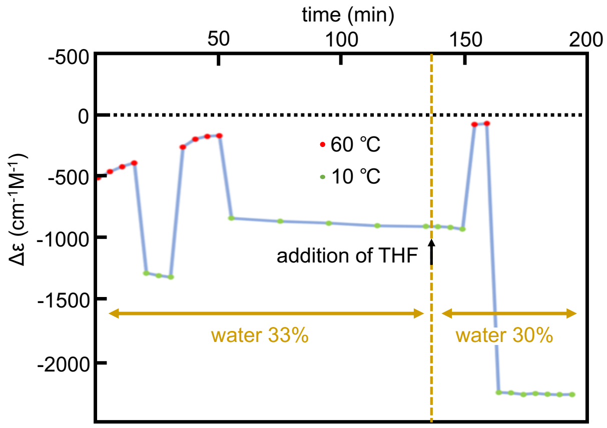

Figure S12. (a) CD and (b) UV-vis spectra of (*M*)-**1**/(*P*)-**2** (1:1) in 33% water-THF (10 μM), which was changed to 30% water-THF by adding THF. (c) The Δε (371 nm)/time profiles are shown by changing the temperature between 10 and 60 °C. (c) is identical with Figure 5a.

<From 30% to 40% experiment>

Solutions of (*M*)-**1** and (*P*)-**2** in 30% water-THF(10 μM) were prepared, and were mixed in 1:1 ratio. The resulted solution was heated at 60 °C for 10 min to form a 2**A** state, cooled to 10 °C, and heated as shown in Figure S13. Then, water was added to make 31, 32, 33, 34, 35, 36, 37, and 38%

water-THF, and the solutions were heated and cooled, which were analyzed by CD and UV-vis. The Δε (369 nm)/time profiles were obtained by changing the temperature and settling the solution at the temperature.

(a)

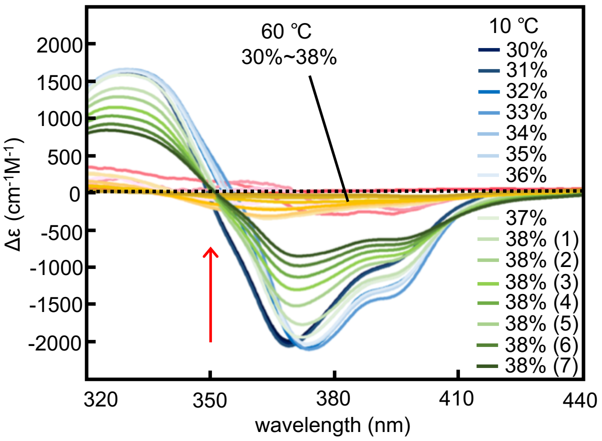

(b)

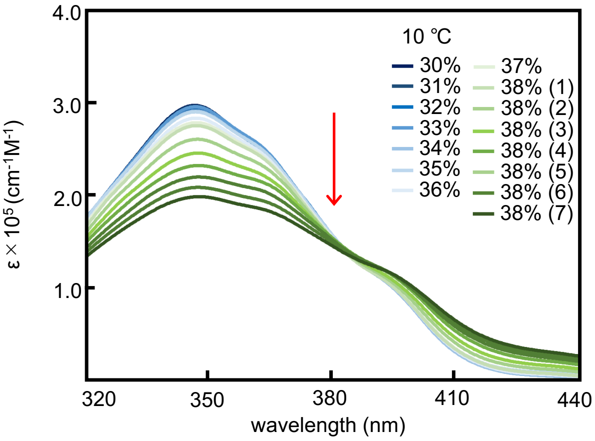

(c)

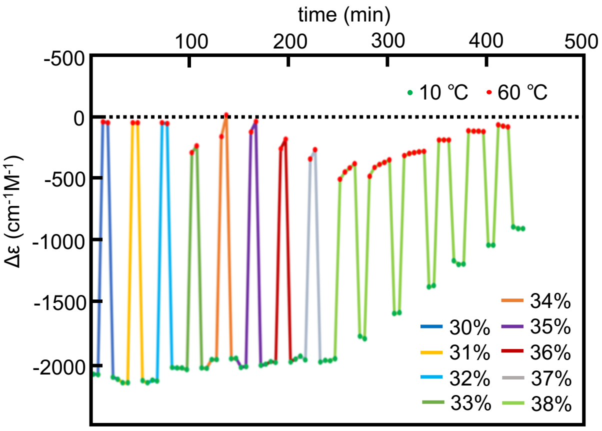

Figure S13. CD (a) and UV-vis (b) spectra of (*M*)-**1**/(*P*)-**2** (1:1) in 30% water-THF (10 μM), which was changed to 31, 32, 33, 34, 35, 36, 37 and 38% water-THF by adding water. The numbers of heating and cooling cycles are shown in parentheses. (c) The Δε (369 nm)/time profiles are shown changing the temperature between 10 and 60 °C. (c) is identical with Figure 5b.

<From 30% to 40% to 33% experiment>

Solutions of (*M*)-**1** and (*P*)-**2** in 30% water-THF (10 μM) were prepared, and were mixed in 1:1 ratio. The resulted solution was heated to 60 °C for 10 min to form a 2**A** state, cooled to 10 °C, and heated as shown in Figure S14. Then, water was added to make 40 and 33% water-THF, which were analyzed by CD and UV-vis. The Δε (372 nm)/time profiles were obtained by changing the temperature and settling the solution at the temperature.

(a)

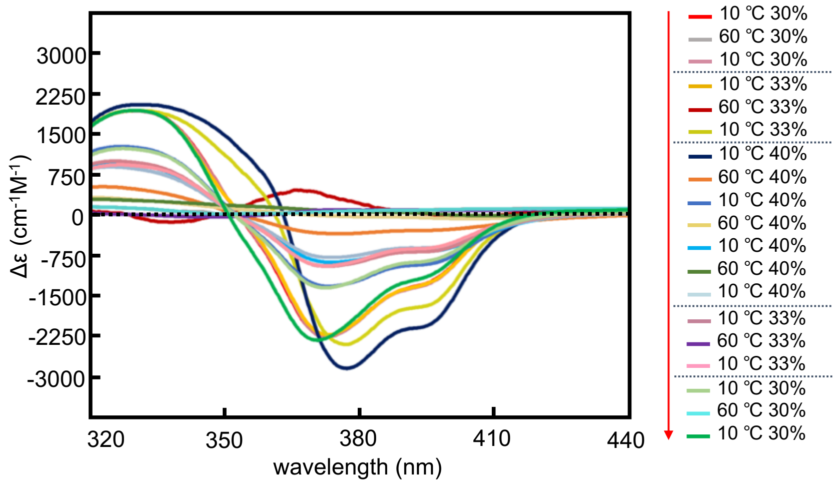
3000
2250
1500
750
0
-750
-1500
-2250
-3000
Δε (cm⁻¹M⁻¹)
320
350
380
410
440
wavelength (nm)
10 °C 30%
60 °C 30%
10 °C 30%
10 °C 33%
60 °C 33%
10 °C 33%
10 °C 40%
60 °C 40%
10 °C 40%
60 °C 40%
10 °C 40%
60 °C 40%
10 °C 40%
10 °C 33%
60 °C 33%
10 °C 33%
10 °C 30%
60 °C 30%
10 °C 30%

(b)

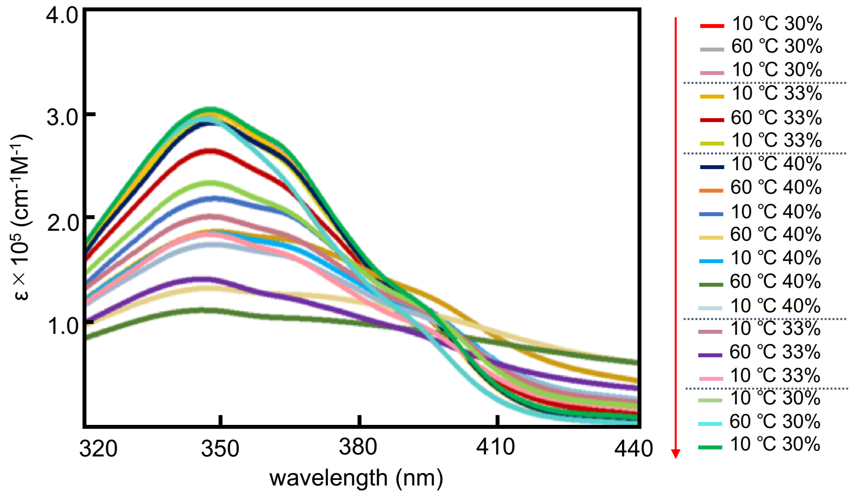
4.0
3.0
2.0
1.0
ε × 10⁵ (cm⁻¹M⁻¹)
320
350
380
410
440
wavelength (nm)
10 °C 30%
60 °C 30%
10 °C 30%
10 °C 33%
60 °C 33%
10 °C 33%
10 °C 40%
60 °C 40%
10 °C 40%
60 °C 40%
10 °C 40%
60 °C 40%
10 °C 40%
10 °C 33%
60 °C 33%
10 °C 33%
10 °C 30%
60 °C 30%
10 °C 30%

(c)

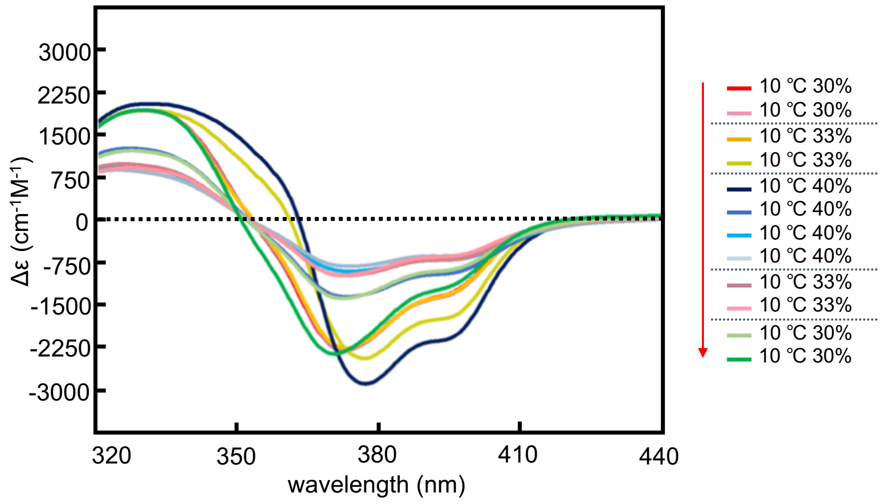
3000
2250
1500
750
0
-750
-1500
-2250
-3000
Δε (cm⁻¹M⁻¹)
320
350
380
410
440
wavelength (nm)
10 °C 30%
10 °C 30%
10 °C 33%
10 °C 33%
10 °C 40%
10 °C 40%
10 °C 40%
10 °C 40%
10 °C 33%
10 °C 33%
10 °C 30%
10 °C 30%

(d)

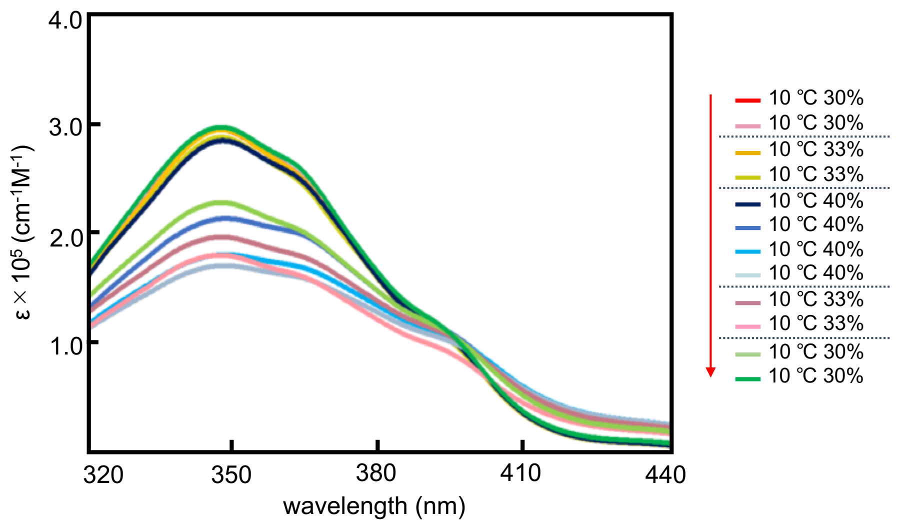

(e)

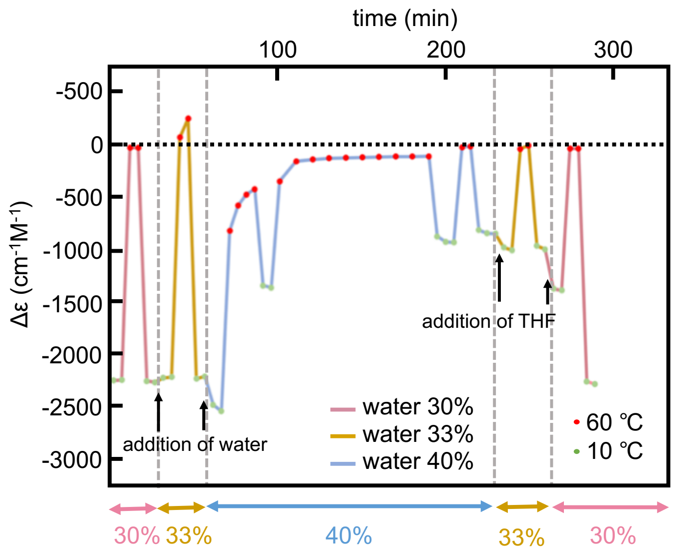

Figure S14. CD (a) and UV-vis (b) spectra of (*M*)-**1**/(*P*)-**2** (1:1) in 30% water-THF (10 μM), which was changed to 33, 40, and 33% water-THF by adding water or THF. (c) and (d) are expansion of (a) and (b), respectively. (e) The Δε (372 nm)/time profiles is identical with Figure 5c.

5. Constant rate temperature change experiment

Solutions of (*M*)-**1** and (*P*)-**2** (1:1) in 30, 33, and 40% water-THF (10 μM) were prepared, and were mixed in 1:1 ratio. The resulted solutions were heated and cooled between 60 and 10 °C at the constant rate of 1 or 0.5 °C/min, the process of which was shown by Δε/temperature profiles.

<25% water-THF solution >

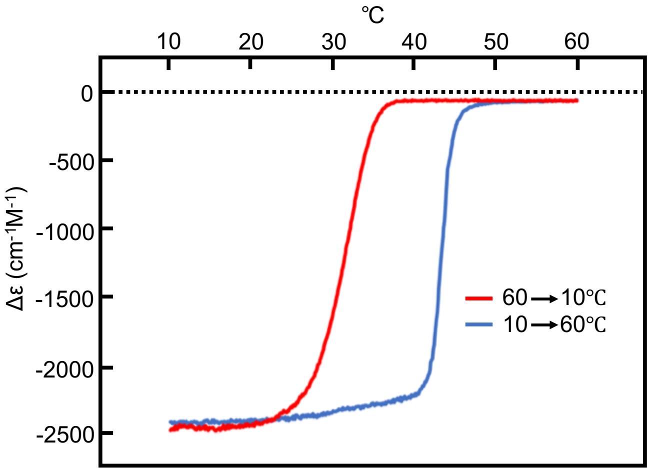

Figure S15. The Δε (371 nm)/temperature profiles of (*M*)-**1**/(*P*)-**2** (1:1) in 25% water-THF (10 μM) under temperature change rate of 1 °C/min

<30% water-THF solution >

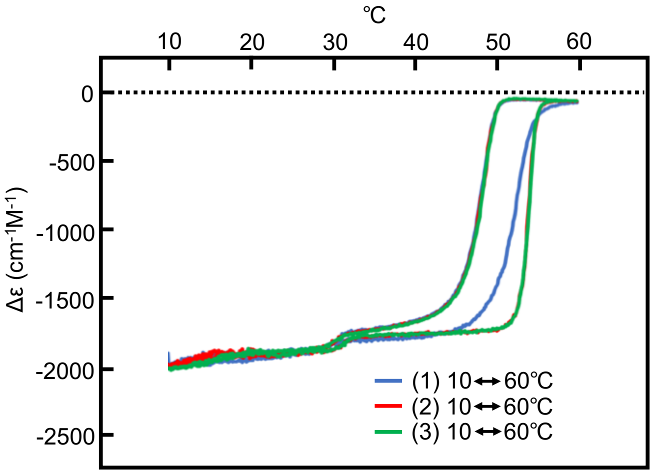

Figure S16. The Δε (375 nm)/temperature profiles of (*M*)-**1**/(*P*)-**2** (1:1) in 30% water-THF (10 μM) under temperature change rate of 1 °C/min. The numbers of heating and cooling cycles are shown in parentheses.

<33% water-THF solution >

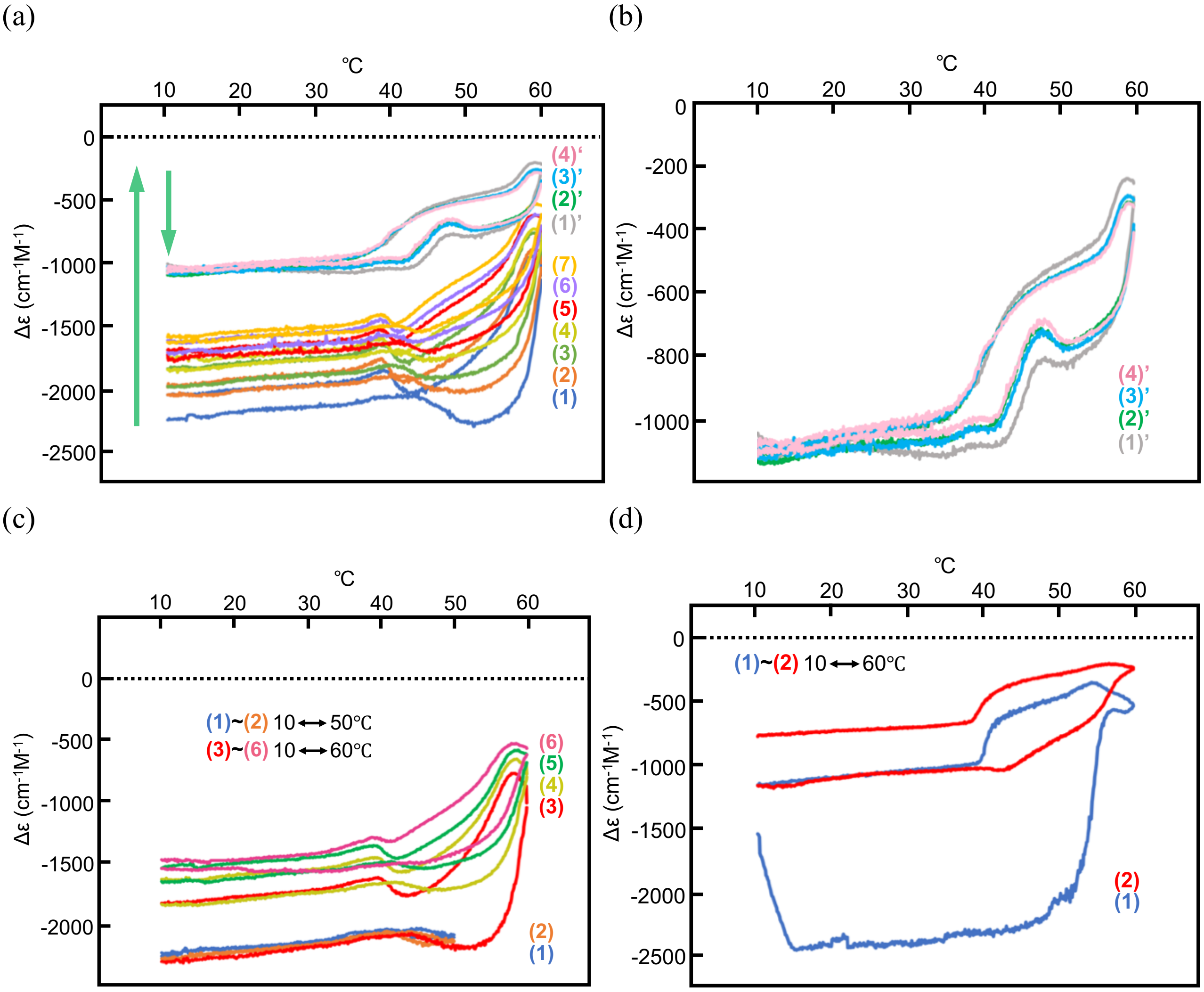

Figure S17. (a) The Δε (375 nm)/temperature profiles of (*M*)-**1**/(*P*)-**2** (1:1) in 33% water-THF (10 μM) under temperature change rate of 1 °C/min. The numbers of heating and cooling cycles are shown in parentheses as (1)-(7). The solution was six times repeatedly heated (60°C, 5 min) and cooled (10°C, 5 min) (data not shown). Then, the solution was subjected to constant rate temperature change experiment at the rate of 1 °C/min providing (1)'-(4)'. (b) An expansion of (1)'-(4)' in (a). (c) Another experiment providing (1)-(6), and (d) its expansion of (1) and (2) are shown.

<40% water-THF solution >

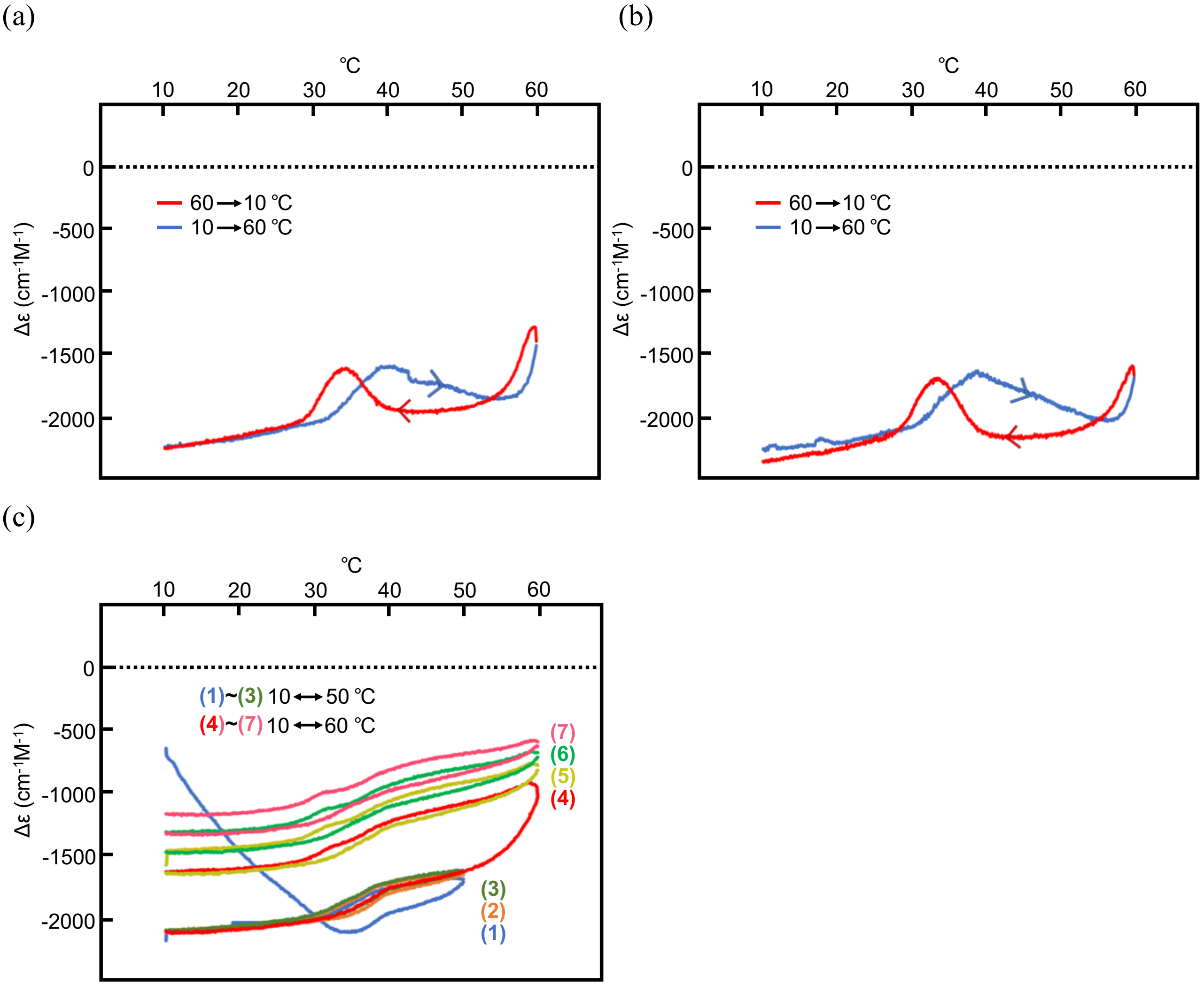

Figure S18. The Δε (369 nm)/temperature profiles of (*M*)-**1**/(*P*)-**2** (1:1) in 40% water-THF (10 ΔμM) under temperature change rate of 1 °C/min (a) and 0.5 °C/min (b). The numbers of heating and cooling cycles are shown in parentheses. (c) Repeated cycles of experiment under temperature change rate of 1 °C/min.

6. References

S1. N. Saito, K. Kanie, M. Matsubara, A. Muramatsu, and M. Yamaguchi, *J. Am. Chem. Soc.*, **2015**, *137*, 6594-6601.

S2. H. Sugiura, Y. Nigorikawa, Y. Saiki, K. Nakamura, and M. Yamaguchi, *J. Am. Chem. Soc.*, **2004**, *126*, 14858-14864.
